# Balanced carbohydrate ratios are associated with improved diet quality in Australia: A nationally representative cross-sectional study

**DOI:** 10.1371/journal.pone.0253582

**Published:** 2021-07-09

**Authors:** Michelle Blumfield, Andrew McConnell, Tim Cassettari, Peter Petocz, Molly Warner, Vanessa Campos, Kim-Anne Lê, Kaori Minehira, Skye Marshall, Flavia Fayet-Moore

**Affiliations:** 1 Department of Science, Nutrition Research Australia, Sydney, New South Wales, Australia; 2 Department of Translational Science, Nutrition Research Australia, Sydney, New South Wales, Australia; 3 Nestlé Institute of Health Sciences, Nestlé Research, Société des Produits Nestlé S.A., Lausanne, Switzerland; 4 Nestlé Research, Société des Produits Nestlé S.A., Vers-chez-les-Blanc, Lausanne, Switzerland; 5 Bond University Nutrition and Dietetics Research Group, Faculty of Health Sciences and Medicine, Bond University, Gold Coast, Queensland, Australia; 6 Nutrition Research Australia, Sydney, New South Wales, Australia; Higher Institute of Applied Sciences and Technology of Gabes University of Gabes, TUNISIA

## Abstract

**Background:**

Carbohydrate quality influences major health outcomes; however, the best criteria to assess carbohydrate quality remain unknown.

**Objective:**

The objectives were to: i) evaluate whether a diet that meets a carbohydrate ratio (simple, modified or dual ratio) is associated with higher nutrient intakes and diet quality, and ii) model the impact of substituting carbohydrate foods that meet the proposed ratios in place of foods that do not, on nutrient intakes.

**Design:**

A secondary analysis of cross-sectional data from the 2011–12 Australian National Nutrition and Physical Activity Survey.

**Participants/Setting:**

National data from participants aged 2 years and older (n = 12,153).

**Main outcome measures:**

Ratios were defined as (i) simple ratio, 10:1 (10g carbohydrate:≥1g dietary fiber); (ii) modified ratio, 10:1:2 (10g carbohydrate:≥1g dietary fiber:≤2g free sugars); and (iii) dual ratio, 10:1 & 1:2 (10g carbohydrate:≥1g dietary fiber & ≤2g free sugars per 1g dietary fiber). Ratios were compared to nutrient intakes obtained via automated multiple-pass 24-hour dietary recall and diet quality calculated using the Australian Healthy Eating Index.

**Statistical analyses performed:**

Substitution dietary modelling was performed. Data were analyzed using paired and independent sample t-tests.

**Results:**

Ratio adherence was highest for simple (50.2% adults; 28.6% children), followed by dual (40.6% adults; 21.7% children), then modified (32.7% adults; 18.6% children) ratios. Participants who met any ratio reported higher nutrient intake and diet quality compared to those who failed to meet the respective ratio (*P* < .001 for all), with the greatest nutrient intakes found for those who met modified or dual ratios. Dietary modelling improved nutrient intakes for all ratios, with the greatest improvement found for the dual ratio.

**Conclusions:**

All carbohydrate ratios were associated with higher diet quality, with a free sugars constraint in the dual ratio providing the greatest improvements.

## Introduction

Carbohydrate (CHO) quality is known to highly influence major health outcomes [1]. Compelling evidence from randomized controlled trials, prospective studies, and dose-response effects suggest a causal relationship between high dietary fiber and a reduced risk of non-communicable diseases [1]. High intakes of dietary fiber, which is the non-digestible edible component of plants, can reduce the risk of cardiometabolic risk factors, including obesity, high cholesterol, high blood pressure and insulin resistance [1–4]. Dietary fiber has also been positively associated with improved gastrointestinal function, and the prevention of diverticular disease and colorectal cancer [5–7]. Conversely, high dietary intake of free sugars promotes adverse health outcomes including dental carries and weight gain [8]. Reflecting the importance of CHO quality for human health, as well as a high prevalence of diets characterized by low CHO quality internationally [9–14], in 2015 the World Health Organization (WHO) recommended diets contain less than 10% of total energy from free sugars, with greater health benefits achievable when free sugars are reduced to less than 5% of total energy [15]. Therefore, developing a metric to assess dietary CHO quality may be an important strategy to monitor nutritional-related health risk for populations and assist in identifying, guiding and promoting healthier CHO food choices and products.

Many different indicators exist to assess CHO quality of foods such as the amount of dietary fiber, wholegrains, free sugars, nutrient density, glycemic index (GI), and glycemic load (GL) [1, 16–18]. These markers can differ in how accurately they represent a high-quality food choice and can be misunderstood by consumers [19–21]. While it is mandatory in some countries to report the total CHO, sugars and dietary fiber content of packaged foods and beverages [22–25], research confirms that consumers find nutrition information on packaging challenging to interpret and are thus rarely used effectively [19–21]. Different criteria have been proposed to help consumers to identify healthier carbohydrate foods based on their wholegrain content. However, there is no universally accepted definition for a wholegrain product [26], and some products that feature a front-of-package wholegrain claim can include a range of wholegrain and refined grain contents in conjunction with higher calories and added sugars [21]. The GI metric has a physiological basis and may offer a simpler alternative to reading nutrition information on packaging; however, there is controversy surrounding the effectiveness of using GI to guide food choices [27, 28], as low GI foods may be high in fructose and/or saturated fat (e.g. fruit muffin) and conversely, high GI foods may also be rich in micronutrients (e.g. boiled potatoes eaten with skin) [1, 29]. Systematic reviews indicate the certainty of evidence for a relationship between GI and GL metrics and cardiometabolic health is of very low to low quality [1], while others found probable to convincing evidence for strength and causality of relationships between GL and type 2 diabetes [30, 31]. The GI and GL metric may not be readily available to manufacturers or consumers for labelling and decision purposes, respectively. Therefore, due to the public health impact of CHO quality [1], the development of an alternative metric to measure CHO quality, considering total CHO, dietary fiber, and free sugars, is warranted.

Based on dietary recommendations and scientific evidence, three simplified ratios to express a balanced CHO composition of foods have been previously developed [32]. A 10:1 ratio, of at least 1g of dietary fiber per 10g of total CHO, was developed based on the composition of whole wheat. The American Heart Association [33] has recommended the 10:1 ratio as it has been linked to foods with higher dietary fiber and lower free sugars, sodium, and trans fats, without increased energy [21], as well as decreased risk of type 2 diabetes in adults [34]. To further improve the 10:1 ratio, a 10:1:2 ratio, of at least 1 g of dietary fiber per 10g of CHO and less than 2g of free sugars, was developed based on an interpretation of the WHO and Scientific Advisory Committee on Nutrition guidelines [15, 32, 35, 36]. Finally, a 10:1 & 1:2 ratio, of at least 1g of dietary fiber per 10g of CHO and per each 1g of dietary fiber less than 2g of free sugars, limits free sugars intake based on dietary fiber content rather than total CHO was also tested [32, 35]. These carbohydrate ratios were named the simple (10:1), modified (10:1:2) and dual (10:1 & 1:2) ratios. Previous work has applied these ratios at the food product level [32], and demonstrated that products that meet these ratios have higher nutritional quality [32, 37]. No research has yet established if these ratios are associated with overall diet quality, thereby reducing translation to public health and clinical practice.

Therefore, in pediatric and adult populations, the aims of this study were to: i) evaluate whether a diet that meets one of three CHO ratios (simple ratio, modified ratio, dual ratio) is associated with higher nutrient intakes and overall diet quality, and ii) model the impact of substituting CHO foods that meet the proposed ratios in place of foods that do not. We hypothesize that Australian diets that include a free sugars constraint [i.e., *modified ratio* (10:1:2) or *dual ratio* (10:1 and 1:2)] will report the most favorable improvements in diet quality compared to diets that do not satisfy these metrics.

## Materials and methods

This study was reported according to the Strengthening the Reporting of Observational Studies in Epidemiology (STROBE) checklist for cross-sectional studies [38]. The interview components of the survey were conducted under the Census and Statistics Act 1905; thus, ethics approval was not required. Informed consent was sought from adults and parents/legal guardians of children through completion of a written consent form. Individuals were approached by mail explaining the study and how information would be kept confidential in accordance with the Census and Statistics Act 1095. Individuals willing to participate completed informed consent forms and kept a copy for their records.

### Survey methodology

This study was a secondary analysis of cross-sectional data collected in the 2011–12 National Nutrition and Physical Activity Survey (NNPAS). The NNPAS is a nationally representative survey carried out by the Australian Bureau of Statistics (ABS) that forms part of the 2011–2013 Australian Health Survey [39]. Data were weighted to represent the Australian population with weightings provided by the ABS. Detailed dietary information and physical activity data were collected for the NNPAS during face-to-face interviews by trained interviewers. An automated multiple-pass 24-hour dietary recall method was used to capture all foods and beverages consumed by respondents within the 24 hours prior to the interview day. For children aged 2–14 years, an adult was interviewed on the child’s behalf, although children aged 6–14 years were invited to also participate in the interview. Data were collected from 9,341 adults aged 19 years and over, and 2,812 children aged 2–18 years who completed the dietary assessment. Although approximately 65% participants were assessed for dietary intake on a subsequent day, to maximize the sample size and ensure it was nationally representative, this study only used dietary intake data from the initial dietary intake assessment. Nutrient analysis was performed using the survey specific 2011–13 Australian Food and Nutrient Database (AUSNUT) [40]. Further survey details including sampling methodology and response rates are available in the Australian Health Survey: Users’ Guide, 2011–13 [41].

### Carbohydrate quality ratios

Three CHO quality ratios were examined: the simple ratio was defined as at least 1g of dietary fiber per 10g of CHOs; the modified ratio referred to at least 1g of dietary fiber and less than 2g of free sugars per 10g of CHOs; the dual ratio was at least 1g of dietary fiber per 10g of CHOs and less than 2g of free sugars per 1g of dietary fiber. These ratios have been recently tested at the food product level and validated as carbohydrate quality metrics [32]. Participants were defined as having ‘satisfied’ or ‘not satisfied’ each ratio based on total daily nutrient intakes.

### Participant charactersitics

Participant characteristics used to describe the sample and interpret the findings included age, sex, anthropometry, and socio-economic status. Physical measurements including weight and height were measured for all respondents by trained interviewers during the face-to-face interview. Interviewers used digital scales to measure weight (maximum 150kg) and a stadiometer to measure height (maximum 210cm) [41]. Participants were encouraged to remove their shoes and any heavy clothing (e.g. jackets) prior to being measured; however, this was voluntary and may not have occurred [41]. Physical measurements were taken once, with a random sample of participants (10%) selected for repeat measurement to ensure the validity of data collected [41]. For children, body mass index (BMI) z-score was calculated using the child’s age, sex, height, and weight, and the WHO growth reference standards for 2–4 and 5-19-year-old children [42]. The standard normal distribution was used to categorize children as: “recommended weight” (<85%), “at risk for overweight” (≥85% to <95%) or “overweight” (≥95%) [43]. For adults, BMI was calculated using the measured weight and height (kg/m^2^), and participants were categorized according to their BMI as underweight (<18.5 kg/m^2^), recommended weight (≥18.5 kg/m^2^, <25 kg/m^2^), overweight (≥25 kg/m^2^, <30 kg/m^2^), or obese (≥30 kg/m^2^) [44]. Socio-economic status was defined by the Socio-Economic Indexes for Areas, a product by the ABS [45] which ranks areas in Australia into quintiles according to relative socio-economic advantage or disadvantage.

### Nutrient intakes

The macronutrients analyzed were energy, protein, total fat, saturated fat, monounsaturated fat, polyunsaturated fat, total CHO, added sugars, free sugars, starch, and dietary fiber. Free sugars were defined as added sugars plus the sugar component of honey and fruit juice, and excluded the naturally occurring sugars in fruits, vegetables, and dairy [15]. The micronutrients analyzed were iron, magnesium, folic acid, zinc, sodium, potassium, and vitamins B1, B2, B3, B6, B12, and E. The other fat-soluble vitamins (A, K) were excluded as CHO-rich foods are not known to be a source of these vitamins and vitamin D data were not available in food composition tables. Energy-standardized nutrient intakes were determined by calculating the daily nutrient intake per 239 kcal (1000 kJ) of energy intake for each participant.

### Healthy Eating Index for Australian adults

Overall diet quality for adults was quantified by the validated Healthy Eating Index for Australian Adults (HEIFA-2013) [17]. The HEIFA-2013 was modelled on the Healthy Eating Index (HEI) [46] but modified for the Australian population to reflect the updated Dietary Guidelines for Australian Adults and the Australian Guide to Healthy Eating [47]. The HEIFA-2013 is calculated based on an 11-component system of five core food groups (vegetables, fruits, grains, dairy and dairy alternatives, and meat and meat alternatives), three negative nutrients (fats, added sugars, and sodium), water intake, and alcohol intake. The components also included discretionary foods and beverages, which are those high in saturated fat and/or added sugars, added salt, or alcohol [47].

The HEIFA-2013 was used to give Australian adults a score between 0 (worst diet quality) and 100 (best diet quality). A score from 1 to 10 was allocated to the following nine components: five core food groups, discretionary foods and beverages, and the negative nutrients saturated fat, added sugar and sodium. A score from 0 to 5 was allocated to the two components water and alcohol. Scores for each component corresponded to how close the respondent was to the recommended number of servings based on their age and sex. HEIFA-2013 is not validated for use in children and adolescents and was therefore applied to those aged ≥18-years only [17].

### Substitution model

Diet modelling analyses were conducted to identify the impact of consuming carbohydrate-rich foods that satisfy the CHO quality ratios on estimated nutrient intakes. Foods in the AUSNUT database were given unique 8-digit codes by the ABS, which are subdivided into major (2-digit), sub-major (3-digit), and minor (5-digit) food groups. Only foods from the two CHO-based major food groups were substituted: cereals and cereal products, and cereal based products and dishes [40]. The ‘cereal and cereal products’ major food group included grains, flours, bread and bread rolls, breakfast cereals, plain pasta, noodles and rice, while the ‘cereal based products and dishes’ food group included biscuits, cakes, pastries, pies, dumplings, pizza, hamburgers, hot dogs, and pasta and rice mixed dishes [40]. There were 1,241 CHO-based foods consumed by survey participants, and each food was classified based on its nutrient profile as either satisfying or not satisfying each of the three CHO quality ratios. For each food that did not satisfy a ratio, the ‘closest’ food was substituted in the model. To determine the closest food, food pairs were given a ‘closeness’ score from 1 to 3 as follows: if the pair of foods were in the same minor (5-digit) food group the closeness score was 1; if they were in the same sub-major (3-digit) food group but different minor group, the score was 2; if they were in the same major (2-digit) food group but different sub-major food group, the score was 3. Where possible, foods that did not meet a ratio were substituted with foods that did which had a closeness score of 1. If there were no ‘1’s, a food with a closeness score of 2 was substituted, and if there were no ‘2’s a food with a score of 3 was substituted.

In cases with more than one ‘closest’ food, the substitution food was selected at random. The randomness was weighted according to the popularity of the foods. For example, if there were two foods (A and B) both with a closeness of 1, if eight participants in the NNPAS consumed food A and four consumed food B, food A would have a 67% chance of being selected and food B a 33% chance. The above substitutions were applied to all survey participants, regardless of whether the participant satisfied the CHO quality ratio in their daily intake, i.e. if a participant met the simple ratio but consumed a CHO-based cereal product that did not satisfy the simple ratio, that product was still substituted in the model.

### Statistical analyses

Prevalence of children and adults meeting each of the three CHO quality ratios was determined among the entire sample, and broken down by age group, sex, BMI category, and socio-economic status. Mean and standard error of daily energy intake was calculated for adults and children, as well as mean and standard error of energy-standardized nutrient intakes (intake per 239 kcal [1000 kJ]). Mean and standard error of each component and the total score of the HEIFA-2013 was determined for adults. The mean and standard error of daily energy and nutrient intakes was calculated for the three substitution models among children and adults.

Independent sample t-tests were used to interpret the differences between nutrient intakes and HEIFA-2013 scores among those who did and did not meet each CHO quality ratio. Paired sample t-tests were used to examine the differences between modelled and reported data. Chi-squared tests were used to investigate association between categorical or ordinal variables. The statistical package IBM SPSS version 26.0 was used for all statistical analyses [48]. Due to the large sample size (n = 12,153) and the number of tests, p-values <0.001 were treated as significant. All variables and data analyses performed were confirmed for accuracy by an independent statistician.

## Results

The ratio met by most participants was the simple ratio (50.2% of adults, 28.6% of children), followed by the dual ratio (40.6% of adults, 21.7% of children), then the modified ratio (32.7% of adults, 18.6% of children).

### Carbohydrate quality ratios according to participant characteristics

Among adults, approximately 10% more females than males satisfied each ratio. In children, girls also satisfied each ratio more than boys, but the difference between sexes was only 1 to 2% (Table 1). More older adults satisfied each ratio than younger adults, where 40 to 64% of those 71+ year-olds met each ratio compared to only 22 to 36% of 19- to 30-year-olds. Among children, more 2- to 3-year-olds (26 to 38%) met each ratio than 14- to 18-year-olds (13 to 23%). There were no clinically relevant differences in those meeting the CHO ratios according to BMI category for adults and children except for underweight adults in which 10–15% fewer participants met each ratio (Table 1). Ratio adherence was greatest for adults and children in the highest quintile for socio-economic status (Table 1). Having an adult health condition (e.g., high cholesterol, hypertension and diabetes) was associated with achieving the simple ratio (p<0.001 for all), while having high cholesterol or hypertension was associated with achieving the modified ratio (p<0.001 for all). More adults and children met each CHO ratio at the dinner eating occasion (41 to 52%; p<0.001) than any other eating occasion (15 to 37%; p<0.001; Table 2 online only).

**Table 1:** Characteristics of participants in the 2011–12 Australian National Nutrition and Physical Activity Survey (NNPAS), by carbohydrate quality ratio.

Characteristics	Total	Simple Ratio^a^		Modified Ratio^b^		Dual Ratio^c^	
	*n*	*n*	*%*	*n*	*%*	*n*	*%*
	**Adults**						
**Age (y)**							
19–30	2160	783	36.3	482	22.3	613	28.4
31–50	3490	1682	48.2	1099	31.5	1351	38.7
51–70	2680	1582	59.0	1067	39.8	1318	49.2
71+	1010	645	63.8	404	40.0	505	50.0
**Sex**							
Male	4613	2059	44.7	1329	28.8	1624	35.2
Female	4728	2633	55.7	1723	36.5	2163	45.8
**Socioeconomic status, IER quintile**							
1	1688	809	47.9	530	31.4	644	38.1
2	1878	891	47.5	605	32.3	729	38.9
3	1925	945	49.1	608	31.6	766	39.8
4	1774	926	52.2	578	32.6	738	41.6
5	2077	1121	54.0	731	35.2	910	43.8
**BMI (kg/m**^**2**^**)**^**d**^							
Underweight (< 18.5)	144	51	35.3	30	20.7	35	24.1
Normal (18.5–24.9)	2833	1393	49.2	898	31.7	1113	39.3
Overweight (25.0–29.9)	2912	1497	51.4	942	32.4	1198	41.2
Obese (≥30.0)	2102	1012	48.1	669	31.8	831	39.5
**Health conditions**							
High cholesterol	916	525	57.3	350	38.2	430	46.9
Hypertensive disease	1454	849	58.4	563	38.7	687	47.2
Diabetes mellitus	591	379	64.1	289	48.9	322	54.5
	**Children**						
**Age (y)**							
2–3	331	125	37.6	87	26.4	96	29.0
4–8	809	266	32.9	169	20.9	198	24.5
9–13	900	235	26.1	163	18.1	186	20.7
14–18	773	178	23.0	103	13.4	130	16.8
**Sex**							
Male	1435	401	27.9	255	17.8	296	20.6
Female	1377	403	29.2	267	19.4	315	22.8
**Socioeconomic status, IER**^**d**^ **quintile**							
1	489	112	23.0	77	15.8	85	17.4
2	528	151	28.6	94	17.8	115	21.9
3	594	165	27.8	116	19.5	131	22.1
4	520	155	29.9	98	18.9	113	21.6
5	681	220	32.3	137	20.1	166	24.3
**BMI z-score**^**d**^							
Normal (< 85%)	1591	454	28.6	297	18.7	347	21.8
At risk for overweight (85%-94.9%)	298	84	28.1	59	19.9	66	22.3
Overweight (≥ 95%)	436	131	30.1	87	20.1	106	24.2
**Health conditions**							
High cholesterol	0	0	0	0	0	0	0
Hypertensive disease	3	0	0	0	0	0	0
Diabetes mellitus	6	1	0.1	1	0.2	1	0.2

^a^ Simple ratio (10:1), At least 1g of fibre per 10g of carbohydrates.

^b^ Modified ratio (10:1:2), At least 1g of fibre and no more than 2g of free sugars per 10g of carbohydrates.

^c^ Dual ratio (10:1 & 1:2), At least 1g of fibre per 10g of carbohydrates and no more than 2g of free sugars per 1g of fibre.

^d^ Body mass index (BMI).

^e^ The Index of Economic Resources (IER) is a measure of wealth. A low score indicates a relative lack of access to economic resources in general. A high score indicates relatively greater access to economic resources in general.

**Table 2:** The proportion of participants in the 2011–12 Australian National Nutrition and Physical Activity Survey (NNPAS) who satisfied each carbohydrate quality ratio, by meal occasion.

Characteristics	Simple Ratio^a^		Modified Ratio^b^		Dual Ratio^c^	
	*n*	*%*	*n*	*%*	*n*	*%*
	**Adults**					
**Meal occasion**						
Breakfast	4214	51.7	3116	38.2	3543	43.5
Lunch	4566	55.1	3968	47.9	4250	51.3
Dinner	5706	64.9	4636	52.7	5125	58.3
Snacks	2518	28.7	1647	18.7	1916	21.8
	**Children**					
**Meal occasion**						
Breakfast	813	31.7	611	23.8	681	26.5
Lunch	933	36.5	811	31.7	864	33.8
Dinner	1386	51.6	1111	41.3	1213	45.2
Snacks	668	24.5	417	15.3	489	17.9

^a^ Simple ratio (10:1), At least 1g of fibre per 10g of carbohydrates.

^b^ Modified ratio (10:1:2), At least 1g of fibre and no more than 2g of free sugars per 10g of carbohydrates.

^c^ Dual ratio (10:1 & 1:2), At least 1g of fibre per 10g of carbohydrates and no more than 2g of free sugars per 1g of fibre.

### Association of carbohydrate quality ratios and nutrient intakes

Adults who satisfied any CHO quality ratio had lower energy, total sugars and saturated fat intake but higher protein, monounsaturated and polyunsaturated fatty acids, and dietary fiber than adults who did not satisfy the ratios (p<0.001; Table 3). Adults who satisfied either the simple ratio or the dual ratio also had a higher energy-standardized intake of total fat compared to those that did not achieve the respective ratios (p<0.001). Starch was lower among those who met the simple ratio than those that did not (p<0.001), higher among those who met the modified ratio (p<0.001), and no different among those who met the dual ratio. Micronutrient intakes among adults who satisfied each of the CHO ratios were higher for all other micronutrients compared to those who did not meet each of the respective ratios (p<0.001), except for sodium and vitamin B12. Intakes of sodium were not statistically different when comparing those who did and did not meet each ratio, while vitamin B12 was higher among those who met the modified and dual ratios but not the simple ratio (p<0.001), despite similarities across ratios.

**Table 3:** Energy-standardised (per 1000kJ) daily macro- and micronutrient intakes for adults in the 2011–12 Australian National Nutrition and Physical Activity Survey (NNPAS) who achieved or did not achieve each carbohydrate quality ratio^a^.

Nutrient	Simple Ratio^b^			Modified Ratio^c^			Dual Ratio^d^		
	Yes	No	*P*^*e*^	Yes	No	*P*^*e*^	Yes	No	*P*^*e*^
Energy (kcal)	1797.56 (11.38)	2248.98 (11.74)	*	1847.64 (13.96)	2182.98 (11.42)	*	1864.70 (12.48)	2215.68 (12.36)	*
	**Per 1000 kcal**								
Protein (g)	47.37 (0.33)	40.91 (0.30)	*	48.70 (0.42)	42.14 (0.26)	*	48.26 (0.37)	41.52 (0.28)	*
Total fat (g)	36.32 (0.3)	35.13 (0.28)	*	35.71 (0.36)	35.73 (0.25)	NS	36.46 (0.33)	35.20 (0.26)	*
Saturated fat (g)	12.63 (0.12)	13.78 (0.12)	*	11.90 (0.14)	13.74 (0.11)	*	12.33 (0.12)	13.99 (0.11)	*
Monounsaturated fat (g)	13.93 (0.08)	12.77 (0.06)	*	13.81 (0.09)	13.10 (0.06)	*	14.01 (0.09)	12.90 (0.06)	*
Polyunsaturated fat (g)	5.95 (0.04)	4.93 (0.04)	*	6.03 (0.05)	5.16 (0.03)	*	6.09 (0.05)	5.00 (0.00)	*
Total carbohydrates (g)	101.58 (0.68)	115.16 (0.76)	*	101.19 (0.89)	112.23 (0.65)	*	100.80 (0.78)	113.72 (0.69)	*
Total sugars (g)	44.21(0.36)	54.25 (0.50)	*	38.42 (0.40)	54.05 (0.41)	*	40.75 (0.37)	54.60 (0.44)	*
Added sugar (g)	14.21 (0.20)	32.90 (0.40)	*	8.66 (0.14)	30.69 (0.33)	*	10.72 (0.15)	32.04 (0.35)	*
Free sugar (g)	17.89 (0.23)	46.46 (0.42)	*	10.82 (0.15)	34.81 (0.34)	*	13.40 (0.17)	36.10 (0.37)	*
Starch (g)	55.83 (0.32)	59.62 (0.37)	*	60.62 (0.41)	56.32 (0.31)	*	58.27 (0.37)	57.63 (0.33)	NS
Fiber (g)	14.74 (0.10)	8.00 (0.06)	*	15.15 (0.14)	9.62 (0.06)	*	15.01 (0.12)	8.57 (0.06)	*
Thiamine (mg)	0.84 (0.01)	0.67 (0.01)	*	0.87 (0.01)	0.69 (0.01)	*	0.86 (0.01)	0.68 (0.01)	*
Riboflavin (mg)	0.95 (0.01)	0.84 (0.01)	*	0.97 (0.01)	0.87 (0.01)	*	0.97 (0.01)	0.86 (0.01)	*
Niacin equivalents (mg)	21.58 (0.15)	18.67 (0.14)	*	22.19 (0.19)	19.24 (0.12)	*	21.45 (0.17)	18.95 (0.13)	*
Vitamin B6 (μg)	0.79 (0.01)	0.67 (0.01)	*	0.81 (0.01)	0.69 (0.01)	*	0.80 (0.01)	0.68 (0.01)	*
Folate equivalents (μg)	326.84 (2.63)	266.34 (2.37)	*	338.74 (3.41)	275.77 (2.05)	*	335.12 (3.00)	270.76 (2.17)	*
Vitamin B12 (μg)	2.26 (0.03)	2.13 (0.03)	NS	2.33 (0.04)	2.15 (0.03)	*	2.31 (0.04)	2.12 (0.03)	*
Vitamin E (μg)	6.00 (0.06)	4.27 (0.04)	*	6.17 (0.08)	4.63 (0.04)	*	6.17 (0.07)	4.42 (0.04)	*
Iron (mg)	6.26 (0.05)	4.58 (0.04)	*	6.44 (0.06)	4.90 (0.03)	*	6.43 (0.05)	4.74 (0.03)	*
Magnesium (mg)	191.05 (1.23)	139.62 (0.98)	*	196.43 (1.61)	149.79 (0.86)	*	195.71 (1.41)	144.86 (0.89)	*
Potassium (mg)	1644.21 (10.17)	1200.98 (8.22)	*	1663.96 (13.20)	1298.67 (7.42)	*	1669.71 (11.67)	1253.16 (7.69)	*
Sodium (mg)	1159.47 (9.72)	1183.64 (9.87)	NS	1172.08 (12.18)	1173.16 (8.49)	NS	1171.05 (10.91)	1173.29 (9.02)	NS
Zinc (mg)	5.95 (0.05)	4.76 (0.04)	*	6.11 (0.06)	4.95 (0.04)	*	6.06 (0.06)	4.87 (0.04)	*

^a^ All values are means; standard error in parentheses.

^b^ Simple ratio (10:1), At least 1g of fibre per 10g of carbohydrates.

^c^ Modified ratio (10:1:2), At least 1g of fibre and no more than 2g of free sugars per 10g of carbohydrates.

^d^ Dual ratio (10:1 & 1:2), At least 1g of fibre per 10g of carbohydrates and no more than 2g of free sugars per 1g of fibre.

^e^ P-values were derived by independent samples t-tests; statistical significance is denoted by * (p<0.001); comparisons are between individuals who achieved and didn’t achieve each ratio.

Among children, energy intake was lower among those who satisfied each CHO ratio compared to those who did not respectively (p<0.001) (Table 4). Children who met any of the CHO ratios had a higher intake of protein, polyunsaturated fatty acids, and dietary fiber; and lower intakes of energy-standardized intakes of saturated fat, total CHOs, and total, added, and free sugars compared to those who did not (p<0.001). There was no difference in intakes of total fat and monounsaturated fatty acids between children who did and did not meet each of the respective ratios, while starch intake was higher among those who met the modified and dual ratios but not the simple ratio (p<0.001). Similar to adults, micronutrient intakes among children were higher in those who satisfied each of the CHO quality ratios compared to those who did not for all micronutrients except sodium and vitamin B12. Sodium intake was lower among children who met the simple ratio than those who did not (p<0.001) but there was no difference in the other ratios, and there was no difference in vitamin B12 intakes across all ratios.

**Table 4:** Energy-standardised (per 1000kJ) daily macro- and micronutrient intakes for children in the 2011–12 Australian National Nutrition and Physical Activity Survey (NNPAS) who achieved or did not achieve each carbohydrate quality ratio^a^.

Nutrient	Simple Ratio^b^			Modified Ratio^c^			Dual Ratio^d^		
	Yes	No	*P*^*e*^	Yes	No	*P*^*e*^	Yes	No	*P*^*e*^
Energy (kcal)	1731.63 (25.05)	1983.53 (17.61)	*	1683.67 (29.97)	1963.05 (16.44)	*	1677.73 (27.13)	1976.32 (16.85)	*
	**Per 1000 kcal**								
Protein (g)	43.56 (0.42)	38.45 (0.25)	*	44.79 (0.54)	38.81 (0.25)	*	44.64 (0.50)	38.61 (0.25)	*
Total fat (g)	34.56 (0.29)	35.41 (0.17)	NS	34.35 (0.38)	35.36 (0.17)	NS	34.45 (0.33)	35.36 (0.17)	NS
Saturated fat (g)	13.83 (0.17)	15.07 (0.13)	*	13.58 (0.21)	14.98 (0.08)	*	13.59 (0.21)	15.03 (0.08)	*
Monounsaturated fat (g)	12.70 (0.14)	12.93 (0.09)	NS	12.67 (0.18)	12.91 (0.08)	NS	12.72 (0.16)	12.90 (0.08)	NS
Polyunsaturated fat (g)	4.95 (0.07)	4.58 (0.05)	*	5.02 (0.09)	4.61 (0.04)	*	5.07 (0.08)	4.58 (0.04)	*
Total carbohydrates (g)	121.27 (0.75)	128.31 (0.46)	*	120.16 (0.92)	127.72 (0.42)	*	120.09 (0.88)	128.01 (0.46)	*
Total sugars (g)	53.55 (0.67)	61.93 (0.46)	*	48.26 (0.75)	62.12 (0.42)	*	49.40 (0.71)	62.34 (0.46)	*
Added sugar (g)	16.61 (0.42)	34.57 (0.46)	*	11.22 (0.29)	33.60 (0.42)	*	12.53 (0.33)	34.12 (0.42)	*
Free Sugars (g)	20.90 (0.46)	39.10 (0.46)	*	13.65 (0.29)	38.53 (0.42)	*	15.60 (0.33)	38.97 (0.42)	*
Starch (g)	66.33 (0.64)	65.36 (0.44)	NS	70.34 (0.81)	64.58 (0.40)	*	69.18 (0.75)	64.65 (0.41)	*
Fiber (g)	15.25 (0.13)	8.91 (0.04)	*	15.57 (0.17)	9.62 (0.08)	*	15.65 (0.17)	9.36 (0.08)	*
Thiamine (mg)	1.11 (0.04)	0.83 (0.00)	*	1.17 (0.04)	0.85 (0.00)	*	1.14 (0.04)	0.84 (0.00)	*
Riboflavin (mg)	1.14 (0.04)	0.98 (0.00)	*	1.18 (0.04)	0.99 (0.00)	*	1.15 (0.04)	0.99 (0.00)	*
Niacin equivalents (mg)	20.12 (0.25)	17.27 (0.13)	*	20.91 (0.33)	17.45 (0.13)	*	20.77 (0.21)	17.34 (0.08)	*
Vitamin B6 (μg)	0.72 (0.00)	0.56 (0.00)	*	0.72 (0.00)	0.58 (0.00)	*	0.73 (0.00)	0.57 (0.00)	*
Folate equivalents (μg)	401.15 (6.82)	325.54 (3.93)	*	413.36 (8.70)	332.13 (3.68)	*	411.43 (7.91)	329.34 (3.77)	*
Vitamin B12 (μg)	2.13 (0.04)	2.03 (0.04)	NS	2.20 (0.04)	2.03 (0.04)	*	2.14 (0.04)	2.03 (0.04)	NS
Vitamin E (μg)	5.27 (0.13)	4.11 (0.04)	*	5.46 (0.17)	4.21 (0.04)	*	5.41 (0.17)	4.17 (0.04)	*
Iron (mg)	6.21 (0.08)	4.79 (0.04)	*	6.31 (0.08)	4.94 (0.04)	*	6.24 (0.08)	4.90 (0.04)	*
Magnesium (mg)	165.71 (1.34)	126.55 (0.63)	*	168.40 (1.72)	130.78 (0.67)	*	169.10 (1.55)	129.05 (0.63)	*
Potassium (mg)	1522.81 (14.90)	1205.89 (7.99)	*	1526.26 (19.04)	1244.34 (7.87)	*	1530.90 (17.61)	1231.50 (7.87)	*
Sodium (mg)	1281.90 (16.15)	1202.32 (10.25)	*	1273.77 (19.46)	1214.23 (9.67)	NS	1278.50 (18.28)	1210.25 (9.83)	*
Zinc (mg)	5.49 (0.08)	4.58 (0.04)	*	5.63 (0.08)	4.67 (0.04)	*	5.55 (0.08)	4.65 (0.04)	*

^a^ All values are means; standard error in parentheses.

^b^ Simple ratio (10:1), At least 1g of fibre per 10g of carbohydrates.

^c^ Modified ratio (10:1:2), At least 1g of fibre and no more than 2g of free sugars per 10g of carbohydrates.

^d^ Dual ratio (10:1 & 1:2), At least 1g of fibre per 10g of carbohydrates and no more than 2g of free sugars per 1g of fibre.

^e^ P-values were derived by independent samples t-tests; statistical significance is denoted by * (p<0.001); comparisons are between individuals who achieved and didn’t achieve each ratio.

### Association of carbohydrate quality ratios and overall diet quality

**Fig 1:**
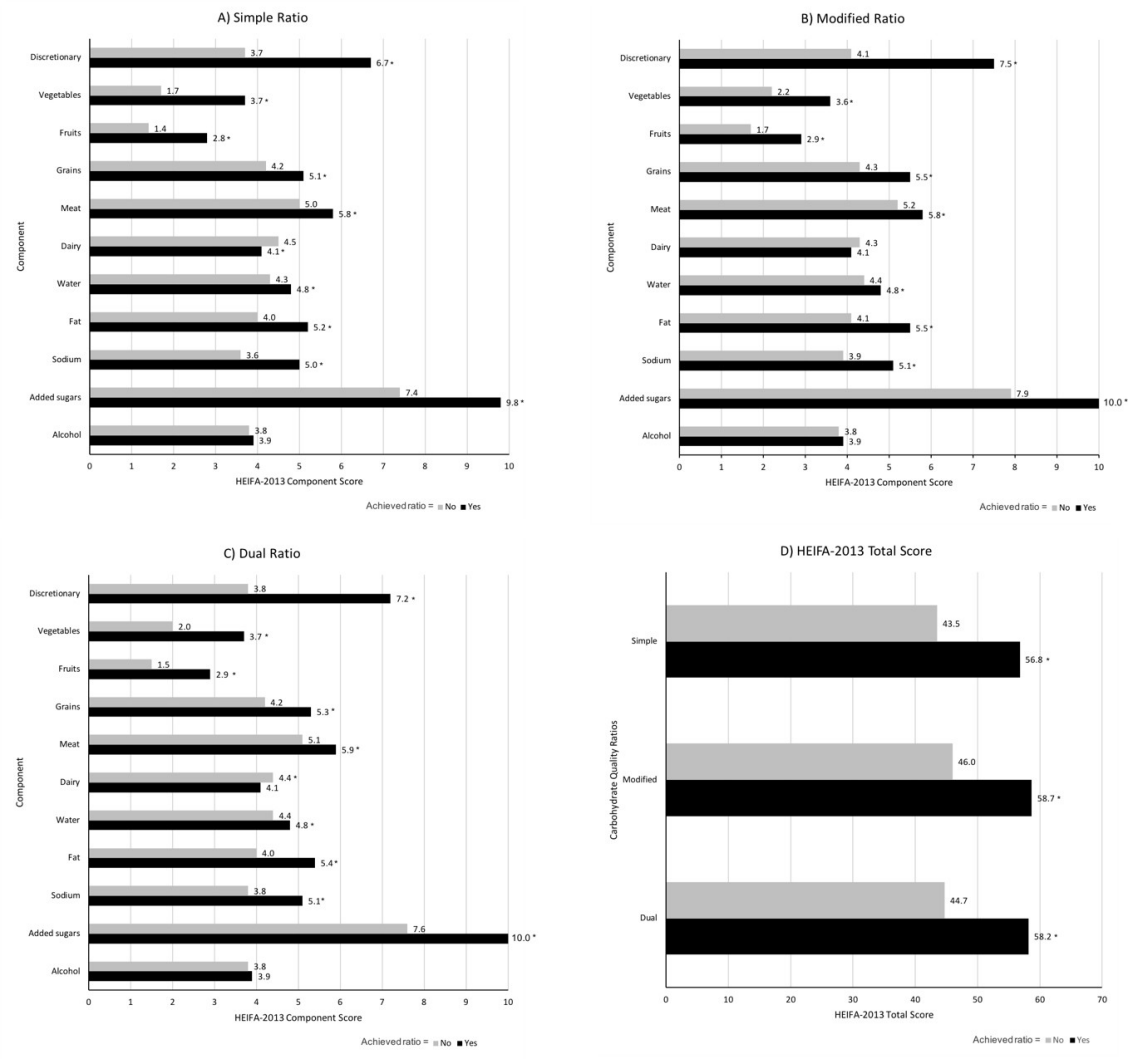
Healthy Eating Index for Australian adults (HEIFA-2013) 11 component scores and total score for adults in the 2011–2012 Australian National Nutrition and Physical Activity Survey (NNPAS) who achieved or did not achieve each carbohydrate quality ratio. (A) Simple ratio (10:1), at least 1g of fiber per 10g of carbohydrates; (B) Modified ratio (10:1:2), at least 1g of fiber and no more than 2g of free sugars per 10g of carbohydrates; (C) Dual ratio (10:1 & 1:2), at least 1g of fiber per 10g of carbohydrates and no more than 2g of free sugars per 1g of fiber; and (D) HEIFA-2013 total scores. *** *P*<0.001 compared to adults who did not achieve the ratio, derived by independent samples t-tests.

For each of the three ratios, adults who satisfied the respective ratio had better overall diet quality than those who failed to meet the ratio (p<0.001); with the greatest difference in HEIFA-2013 scores among those who met the dual ratio (13.5/100 difference), followed by the simple ratio (13.3/100 difference), and the modified ratio (12.7/100 difference) (Fig 1). Total HEIFA-2013 scores were greatest for the modified (58.7 vs. 56.8; p<0.001) and dual ratios (58.2 vs. 56.8; p<0.001) compared to the simple ratio, while no difference was found in total HEIFA-score between the modified and dual ratio (58.7 vs. 58.2; p = 0.080). When comparing the 11 individual components in the index, diet quality among those who satisfied each respective CHO ratio was better for discretionary intake, vegetables, fruits, grains, meat, water, fat, sodium and added sugars. Adults who met the simple ratio and the dual ratio had a lower diet quality score for dairy than those who did not meet the ratio (p<0.001), and there was no difference in dairy diet quality between those who did and did not meet the modified ratio. There was no difference in the alcohol diet quality across all CHO ratios.

### Effect of substitution dietary modelling on nutrient intake

When CHO-based cereal products that did not meet each ratio were substituted by similar foods that did, energy, protein, total fat, monounsaturated and polyunsaturated fatty acids, and dietary fiber intake among adults increased for all ratios, while total carbohydrates decreased for all ratios (p<0.001 for all) (Table 5). Saturated fat increased with substitution in the simple ratio model (p<0.001), decreased in the dual ratio model (p<0.001), and remained unchanged in the modified ratio. Total CHOs, added and free sugars, and starch decreased with substitution for all three models (p<0.001), while total sugars also decreased for the modified and dual ratio models (p<0.001) but did not change in the simple ratio model. Sodium and vitamin B12 decreased and other micronutrients increased in all three ratio models (p<0.001), except for folate and vitamin E. Folate decreased with substitution in the simple and modified ratios (p<0.001), and intakes of vitamin E increased in the simple and dual ratio models (p<0.001).

**Table 5:** Dietary modelling of the impact of substituting carbohydrate foods that meet each carbohydrate quality ratio in place of foods that do not, on nutrient intakes for adults and children.

Nutrient	Unadjusted^a^	Simple Ratio^b^		Modified Ratio^c^		Dual Ratio^d^	
	Mean (SE)	Mean (SE)	*P*^*e*^	Mean (SE)	*P*^*e*^	Mean (SE)	*P*^*e*^
	**Adults**						
Energy (kcal)	2073 (9.0)	2142 (10.0)	*	2114 (10.0)	*	2116 (10.0)	*
Protein (g)	91.0 (0.5)	94.9 (0.5)	*	94.9 (0.5)	*	92.0 (0.5)	*
Total fat (g)	73.8 (0.4)	80.2 (0.5)	*	78.7 (0.5)	*	78.3 (0.5)	*
Saturated fat (g)	27.7 (0.2)	28.9 (0.2)	*	27.7 (0.2)	NS	27.3 (0.2)	*
Monounsaturated fat (g)	28.4 (0.2)	29.8 (0.2)	*	29.8 (0.2)	*	29.6 (0.2)	*
Polyunsaturated fat (g)	11.4 (0.1)	14.0 (0.1)	*	13.8 (0.1)	*	14.0 (0.1)	*
Total carbohydrates (g)	226 (1.0)	219 (1.0)	*	216 (1.0)	*	217 (1.0)	*
Total sugars (g)	103 (1.0)	103 (1.0)	NS	99 (1.0)	*	99 (1.0)	*
Added sugar (g)	50.6 (0.5)	47.9 (0.5)	*	43.5 (0.5)	*	43.7 (0.5)	*
Free sugar (g)	57.8 (0.6)	55.1 (0.6)	*	50.7 (0.5)	*	50.9 (0.5)	*
Starch (g)	118 (1.0)	111 (1.0)	*	112 (1.0)	*	113 (1.0)	*
Fiber (g)	22.9 (0.1)	35.3 (0.3)	*	34.4 (0.3)	*	34.8 (0.3)	*
Thiamine (mg)	1.5 (0.0)	2.6 (0.0)	*	2.6 (0.0)	*	2.6 (0.0)	*
Riboflavin (mg)	1.9 (0.0)	2.1 (0.0)	*	2.1 (0.0)	*	2.1 (0.0)	*
Niacin equivalents (mg)	41.4 (0.2)	55.9 (0.6)	*	56.4 (0.6)	*	56.3 (0.6)	*
Vitamin B6 (μg)	1.5 (0.0)	2.5 (0.0)	*	2.4 (0.0)	*	2.5 (0.0)	*
Folate equivalents (μg)	610 (4.0)	600 (4.0)	*	603 (4.0)	*	608 (4.0)	NS
Vitamin B12 (μg)	4.5 (0.0)	4.5 (0.0)	*	4.5 (0.0)	*	4.5 (0.0)	*
Vitamin E (μg)	10.5 (0.1)	11.0 (0.1)	*	10.5 (0.1)	NS	10.6 (0.1)	*
Iron (mg)	11.1 (0.1)	13.4 (0.1)	*	13.4 (0.1)	*	13.3 (0.1)	*
Magnesium (mg)	339 (2.0)	594 (8.0)	*	591 (8.0)	*	594 (8.0)	*
Potassium (mg)	2912 (14.0)	3426 (21.0)	*	3409 (21.0)	*	3422 (21.0)	*
Sodium (mg)	2431 (15.0)	2371 15.0)	*	2370 (15.0)	*	2383 (15.0)	*
Zinc (mg)	11.0 (0.1)	12.8 (0.1)	*	12.7 (0.1)	*	12.8 (0.1)	*
	**Children**						
Energy (kcal)	1912 (15.0)	1965 (15.0)	*	1919 (15.0)	NS	1923 (15.0)	NS
Protein (g)	75.0 (0.7)	78.4 (0.7)	*	77.6 (0.7)	*	79.2 (0.7)	*
Total fat (g)	69.1 (0.7)	73.4 (0.7)	*	72.0 (0.7)	*	70.4 (0.7)	*
Saturated fat (g)	29.1 (0.3)	29.7 (0.3)	*	28.7 (0.3)	NS	27.4 (0.3)	*
Monounsaturated fat (g)	25.3 (0.3)	25.8 (0.3)	*	25.8 (0.3)	*	25.3 (0.3)	*
Polyunsaturated fat (g)	9.1 (0.1)	11.5 (0.2)	*	11.0 (0.2)	*	11.3 (0.2)	*
Total carbohydrates (g)	239 (2.0)	233 (2.0)	*	226 (2.0)	*	229 (2.0)	*
Total sugars (g)	112 (1.0)	112 (1.0)	*	105 (1.0)	*	106 (1.0)	*
Added sugar (g)	58.0 (0.9)	53.9 (0.9)	*	47.6 (0.8)	*	47.8 (0.8)	*
Free sugars (g)	66.1 (0.9)	62.0 (0.9)	*	55.7 (0.9)	*	56.0 (0.9)	*
Starch (g)	124 (1.0)	120 (1.0)	*	119 (1.0)	*	121 (1.0)	*
Fiber (g)	19.7 (0.2)	31.5 (0.4)	*	30.1 (0.4)	*	30.9 (0.4)	*
Thiamine (mg)	1.6 (0.0)	2.4 (0.0)	*	2.4 (0.0)	*	2.4 (0.0)	*
Riboflavin (mg)	1.9 (0.0)	2.0 (0.0)	*	2.0 (0.0)	*	2.0 (0.0)	*
Niacin equivalents (mg)	33.5 (0.3)	44.6 (0.7)	*	45.0 (0.7)	*	45.1 (0.7)	*
Vitamin B6 (μg)	1.1 (0.0)	1.9 (0.0)	*	1.8 (0.0)	*	1.9 (0.0)	*
Folate equivalents (μg)	625 (7.0)	579 (6.0)	*	569 (6.0)	*	587 (6.0)	*
Vitamin B12 (μg)	3.9 (0.0)	3.8 (0.0)	*	3.8 (0.0)	*	3.8 (0.0)	*
Vitamin E (μg)	8.4 (0.1)	9.0 (0.1)	*	8.1 (0.1)	*	8.6 (0.1)	*
Iron (mg)	9.7 (0.1)	11.8 (0.1)	*	11.6 (0.1)	*	11.6 (0.1)	*
Magnesium (mg)	255 (2.0)	459 (10.0)	*	456 (10.0)	*	461 (10.0)	*
Potassium (mg)	2403 (20.0)	2830 (28.0)	*	2806 (28.0)	*	2828 (28.0)	*
Sodium (mg)	2313 (23.0)	2251 (23.0)	*	2243 (23.0)	*	2253 (23.0)	*
Zinc (mg)	9.2 (0.1)	10.7 (0.1)	*	10.4 (0.1)	*	10.6 (0.1)	*

^a^ Daily nutrient intake for participants in the 2011–12 Australian National Nutrition and Physical Activity Survey (NNPAS).

^b^ Simple ratio (10:1), At least 1g of fibre per 10g of carbohydrates. Modelled data, carbohydrate foods were substituted with similar foods that satisfied the simple ratio.

^c^ Modified ratio (10:1:2), At least 1g of fibre and no more than 2g of free sugars per 10g of carbohydrates. Modelled data, carbohydrate foods were substituted with similar foods that satisfied the modified ratio.

^d^ Dual ratio (10:1 & 1:2), At least 1g of fibre per 10g of carbohydrates and no more than 2g of free sugars per 1g of fibre.

^e^ *P* values were derived by paired samples t-tests; statistical significance is denoted by * (p<0.001). Modelled data, carbohydrate foods were substituted with similar foods that satisfied the dual ratio.

Among children, energy intake increased with substitution according to the simple ratio model (p<0.001) but remained unchanged in the modified and dual ratio models (Table 5). Intakes of protein, total fat, polyunsaturated fatty acids, and dietary fiber increased with substitution, and intakes of CHOs, starch, total, added, and free sugars decreased in all three models (p<0.001). Saturated fat intake increased with substitution in the simple ratio model and decreased in the dual ratio model (p<0.001), while intake of monounsaturated fatty acids increased in the simple and modified models but decreased in the dual model (p<0.001). Dietary intakes of folate, vitamin B12, and sodium decreased, and other micronutrients increased with substitution among children across all three ratio models (p<0.001), except for vitamin E. Vitamin E increased in the simple and dual ratios and decreased in the modified ratio model (p<0.001).

## Discussion

This is the first study to examine if diets that satisfy CHO quality ratios are associated with higher nutrient intake and diet quality. Results indicate that meeting a CHO quality ratio was associated with lower energy intake and higher nutrient composition and, for adults, overall diet quality, with the addition of a free sugars constraint in the dual ratio providing the greatest improvements. Dietary modelling to substitute CHO-based foods to meet each ratio further resulted in higher nutrient intakes, even among participants who originally met the ratios based on total daily nutrient intakes. Currently, only the simple ratio has been associated with health outcomes and for the utility in identifying the most healthful food products for food labelling purposes [21, 34].

This study found that CHO quality in the Australian diet is poor; ≤50% of adults and <30% of children met the CHO quality ratios, aligning with previous findings which have reported the general Australian population does not adhere to dietary guidelines [49–51]. While it is possible that dietary intakes have changed since 2011–12, research indicates that diet quality remains poor [52, 53]. Dietary fiber intakes have been previously reported to be well below the daily target of 25g to 30g as recommended in the Australia and New Zealand Nutrient Reference Values (NRVs) at a median of 20.7g for adults and 18.2g for children in the NNPAS [51, 54]. Further, free sugars intakes of Australians exceed the WHO guidelines of less than 10% total energy (14.7% for adults, 14.9% for children) [15]. Findings confirm that Australians need to improve their CHO quality and may do so by following a diet that meets a CHO quality ratio metric, with the most favorable improvements achieved by utilizing a free sugars constraint (i.e., the modified and dual ratios).

This study contributes novel research examining the association between CHO quality and diet quality indices. Previous studies have focused on the association between GI or GL and nutrient or food group intakes [55–59], where lower GI diets were associated with higher fruit and dairy consumption [56, 60, 61] and higher GL was associated with poorer nutrient intakes [56]. This study suggests that when diets satisfied a CHO quality ratio, improvements in diet quality were largely driven by increased servings of fruit and vegetables and decreased servings of discretionary foods and drinks, which resulted in improvements to added sugars, sodium and fat. Although smaller improvements were also found for grains, meat and water. This finding aligns with the global priority aimed at increasing fruit and vegetable consumption [62], as research shows that discretionary foods often displace fruit and vegetable intakes [63]. Research has also reported that consuming wholegrain cereals in place of discretionary choices can improve overall diet quality [64]. At the food product level, products that met the CHO quality ratios had an improved nutritional profile (e.g. lower energy and higher micronutrients) and were more likely to meet the required thresholds for nutrient profiling systems in the UK and Australia that are used to identify the healthfulness of a product, and to control front-of-package labelling for packaged foods [32]. Therefore, a CHO quality ratio, based on CHO, dietary fiber and free sugars, may provide a simple, standardized approach to meet this global priority to improve diet quality.

It was interesting that the substitution dietary modelling did not improve intakes of folate and vitamin B12. The lack of improvement for folate may be explained by differences in the fortification of food products. In Australia, wheat has mandatory folic acid fortification, but fortification levels are lower in whole meal and multigrain bread varieties, compared with refined bread [65]. Although not mandatory, many processed cereal-foods (i.e. breakfast cereals and bread products made from other cereal flour) are also fortified with folic acid [65, 66], but other CHO rich foods, such as oats, fruit, and some vegetables, are not. There is no intrinsic reason to fortify only highly refined grains, and these and prior findings suggest that, as industry and consumers turn toward healthier whole grain products, fortification strategies should be reconsidered to also include these healthier grain products. The lack of improvement for vitamin B12 may be due to a reduction in cereal-based products and dishes containing ingredients derived from animals (e.g. pizza, lasagna, biscuits, cakes) as well as limitations placed on vitamin B12 fortification in Australia [67]. Therefore, examination of these ratios in other countries is of interest, such as the United States where foods are extensively fortified with vitamin B12 [67].

There is currently no consensus regarding the most reliable measure of CHO quality. All measures have their strengths and limitations [68]. Findings from a series of systematic reviews and meta-analyses have reported the benefits of low GI or GL diets on human health [1, 30, 31]; however in meta-analyses of prospective studies and clinical trials, effect sizes were modest compared to the protective effects of total dietary fiber or wholegrains [1]. Alternatively, in global dose-response meta-analyses of prospective studies, a positive association between both GI and GL were found with increasing incidence of type 2 diabetes [30, 69]. The GI response of a food may change based on ethnicity [70, 71], and large variations can be reported within and between laboratories [72]. CHO quality ratios present an alternative metric that are not influenced by any variability in individual response and have been reported as reliable metrics to identify food products with improved nutritional profile [32]. Substantial evidence further supports the harmful effects of high free sugar intake on health [15], thus reducing consumption of free sugars is highly recommended. Following a CHO quality ratio will also likely reduce exposure to ultra-processed foods (typically high in energy, salt, sugar and fat), which may be causally linked to poor health [73]. Considering current evidence, a CHO quality ratio that combines two indicators of CHO quality (dietary fiber and free sugars) might prove more useful as an overall measure of CHO quality than individual indices on their own.

Limitations of this study relate to its cross-sectional observational nature, which does not allow causality to be inferred, as well as the use of single-day dietary intake data, which decreases relevance to the usual dietary intake of participants. However, the use of one self-reported 24-hour recall to measure dietary intake is strengthened by similarities in the daily mean energy intake reported by women in this representative sample (7,402kJ/day) and the mean daily energy intake reported by women in the Australian Longitudinal Study on Women’s Health, which used a more rigorous method of dietary intake reporting (7,521kJ/day) [74]. Despite being cross-sectional and limited to the Australian context, the NNPAS provides the best available population level nutrient data at this time and offers statistical power. The generalizability of findings may be limited by the age of the data and impact of global events such as COVID-19. Care should also be taken in extrapolating results from the substitution CHO quality models, as these demonstrate proof of concept only and did not consider non-cereal-based CHO rich foods. A complete substitution of all CHO-based foods that do not meet a CHO quality ratio with similar foods that do may not be practical in a real-world setting.

Individual CHO-based foods and their level of processing in the substitution modelling were not examined. This was outside the scope for this study but would be an important next step for future research to better interpret the findings. To translate the findings of this study to public health policies and health proportion strategies, further research regarding the relationship between the modified and dual ratios on the health of children and adults is required. Future research is also required to confirm the suitability of modified or dual ratios on product labelling to help consumers and organizations quickly and accurately identify healthful CHO products and the impact of this on improvement of overall diet quality.

## Conclusions

All carbohydrate ratios were associated with higher diet quality, with a free sugars constraint in the dual ratio providing the greatest improvements. Further research is required to determine if the utility of a CHO quality metric, based on CHO, dietary fiber, and possibly free sugars offers a simple, standardized approach to measure and improve diet quality. Investigations into the relationship between diets that satisfy a CHO quality ratio and the impact on health outcomes as well as translation to public health policies and strategies is also warranted.

## Abbreviations

AUSNUT: Australian Food and Nutrient Database
BMI: body mass index
CHO: carbohydrate
FSANZ: Food Standards Australia New Zealand
GI: glycaemic index
GL: glycaemic load
g: grams
HEI-2010: Healthy Eating Index
HEIFA-2013: Australian Healthy Eating Index
kJ: kilojoules
kg: kilogram
m: meter
μg: micrograms
NNPAS: National Nutrition and Physical Activity Survey
SACN: Scientific Advisory Committee on Nutrition
WHO: World Health Organisation
